# Metabolic adaptations of the tumor macroenvironment and their role in cancer progression and survivorship

**DOI:** 10.1101/gad.353830.126

**Published:** 2026-08-01

**Authors:** Devesh Raizada, Ana P. Gomes

**Affiliations:** 1 Department of Tumor Microenvironment and Metastasis, Moffitt Cancer Center, Tampa, Florida 33612, USA;; 2 College of Arts and Sciences, University of South Florida, Tampa, Florida 33620, USA

**Keywords:** cancer progression, metabolism, tumor macroenvironment

## Abstract

Cancer progression is shaped not only by tumor-intrinsic programs and the local microenvironment but also by profound metabolic rewiring of the host at the organismal scale. Tumors engage in dynamic cross-talk with distant organs, immune and neuroendocrine networks, and systemic nutrient pools, generating a metabolically permissive macroenvironment that fuels tumor growth, supports metastatic dissemination, and undermines therapeutic efficacy. In this review, we synthesize emerging evidence showing how cancer-driven systemic metabolic remodeling reshapes glucose, lipid, and protein metabolism, perturbs immune and hematopoietic homeostasis, and hijacks neuroendocrine circuits governing energy balance and stress responses. We further highlight how these adaptations fuel cellular energetics within tumor cells as well as promote premetastatic niche formation, immune evasion, and therapy resistance, while leaving durable metabolic and inflammatory scars that accelerate biological aging and compromise long-term health in cancer survivors. Together, these findings position tumor–host metabolic cross-talk as a central, targetable axis in cancer biology with broad implications for therapy and survivorship care.

## Abstract (precis)

In this review, Raizada and Gomes discuss the cancer-induced, tumor–host metabolic cross-talk that supports cancer progression. Metabolic remodeling is a central axis in tumorigenesis, with widespread influence on immunity, neuronal signaling and cognition, and aging and survivorship.

Cancer progression emerges from the dynamic integration of tumor-intrinsic genetic programs with a constellation of extrinsic factors that collectively influence the dynamic and adaptive nature of cancer cells (Maman and Witz 2018). While foundational work has established the central roles of oncogenic mutations and the local tumor microenvironment (TME) in governing tumor growth, immune evasion and therapeutic resistance (Hanahan and Weinberg 2011; Elia and Haigis 2021; de Visser and Joyce 2023), it is increasingly evident that tumors exert far-reaching effects that extend beyond the immediate tumor niche. Through bidirectional communication with distant organs and physiological systems, cancers actively reprogram host metabolism, immunity, and neuroendocrine function, thereby reshaping the systemic environment in which tumors evolve.

The TME constitutes the most proximal layer of tumor–host interaction, regulating malignant behavior through soluble signaling molecules, extracellular matrix components (ECM) and direct cellular contacts (Hanahan and Weinberg 2011; Elia and Haigis 2021; de Visser and Joyce 2023). This local niche is further embedded within the tissue-specific context of the organ of origin, where architectural features, stromal composition, vascular organization among other features impose additional constraints on tumor progression (Almagro et al. 2022; de Visser and Joyce 2023). Superimposed on these local influences is a systemic layer of regulation, in which host factors such as age and metabolic health heavily influence disease progression (Altea-Manzano et al. 2025; Lazure and Gomes 2025). Conversely, tumors actively remodel host physiology, engaging distant organs and regulatory networks to establish a permissive macroenvironment that supports tumor growth, facilitates dissemination and undermines treatment efficacy (Al-Zoughbi et al. 2014; Altea-Manzano et al. 2025). Despite their profound clinical relevance, the molecular mechanisms governing tumor-derived systemic remodeling remain poorly defined.

Metabolic reprogramming is now a widely accepted cornerstone of cancer progression, enabling malignant cells to meet the energetic and redox demands of unchecked proliferation while enabling adaptation to fluctuating nutrient and oxygen availability (Faubert et al. 2020; Bergers and Fendt 2021). Although extensive effort has been devoted to defining tumor-intrinsic metabolic pathways and metabolic interactions within the TME, far less attention has been directed toward understanding how tumors interact metabolically with the host at the organismal scale. Tumors can alter metabolism in the macroenvironment by the direct release of signaling molecules (e.g., cytokines, growth factors, hormones) and extracellular vesicles (EVs) as well as by altering nutrient pools across distant tissues (Kokal et al. 1983; Heber et al. 1985; Fong et al. 2015; Cohen et al. 2023; Xu et al. 2024). In parallel, host responses to cancer also generate indirect but equally consequential metabolic effects (Borniger et al. 2018; Burfeind et al. 2020; Hiam-Galvez et al. 2021; Goldman et al. 2023). These bidirectional interactions create self-reinforcing metabolic circuits that extend across tissues and physiological systems.

In this review, we integrate emerging evidence across metabolism, immunology, neurobiology and aging biology to delineate the mechanisms and consequences of tumor–host metabolic cross-talk. We focus on three major themes: (1) how tumors reprogram host metabolism across distant organs, (2) how these systemic adaptations actively promote tumor progression, and (3) the long-lasting effects of cancer history. By reframing cancer as a systemic metabolic disease, we highlight new conceptual paradigms and therapeutic opportunities aimed not only at suppressing tumor growth but also at restoring durable metabolic and physiological resilience.

## Tumor-induced metabolic remodeling of the host macroenvironment

**Figure 1.**
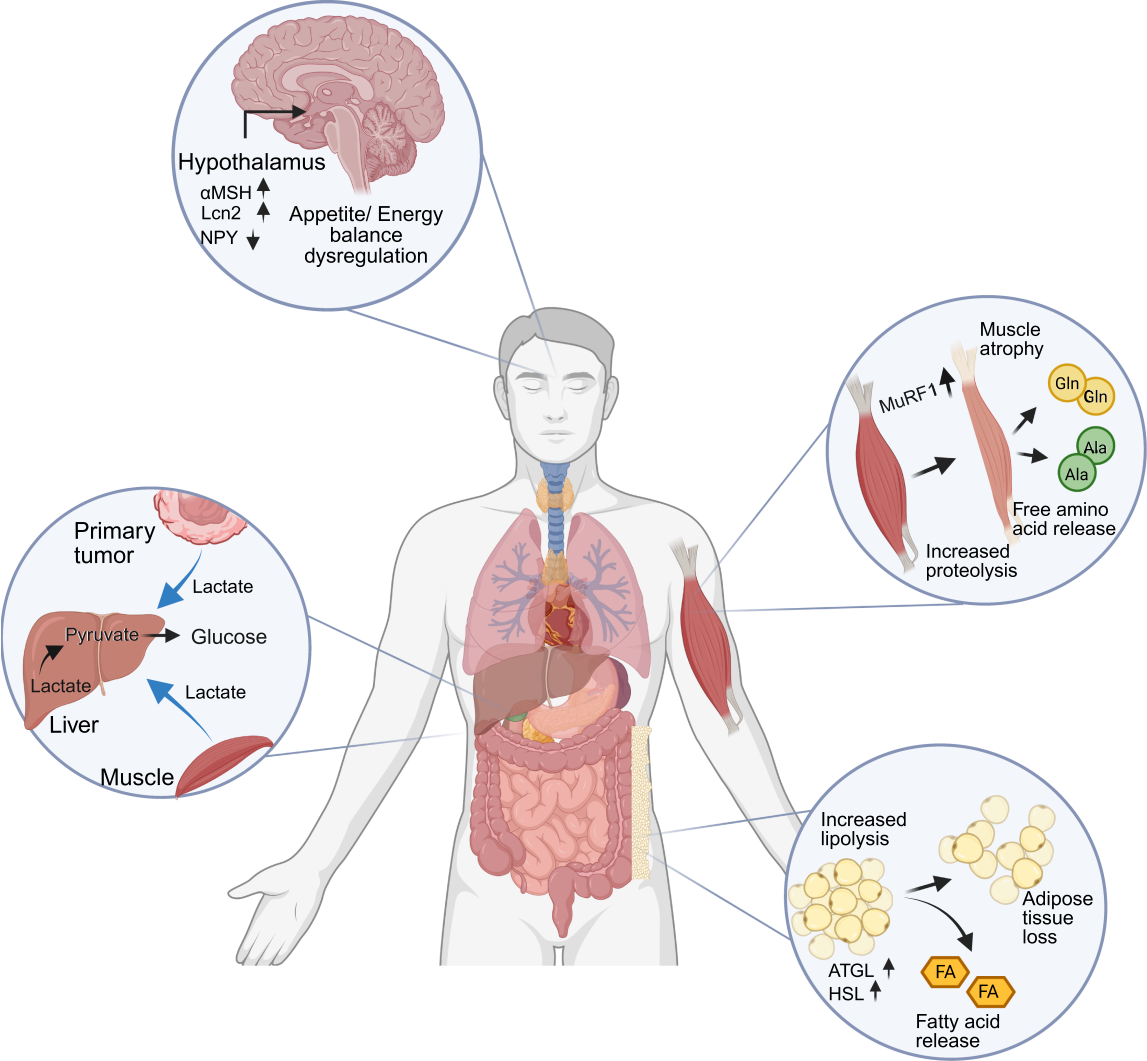
Tumor-induced metabolic alterations across host tissues. Tumors possess the ability to alter appetite and energy balance by regulating hypothalamic signaling in the central nervous system. These changes include hypothalamic release of αMSH, Lcn2 and reduced NPY levels, all of which affect feeding patterns and energetics within the host. Activation of muscle proteolysis by atrophy-related genes like MuRF1 results in the release of amino acids causing major amino acid redistribution systemically. Similarly, tumors also induce adipose tissue wasting through elevated lipolysis driven by lipases like ATGL and HSL promoting systemic fatty acid mobilization. Parallelly, the host liver undergoes increased gluconeogenesis driven by metabolic exchange of gluconeogenic precursors like lactate between peripheral tissues/the tumor itself and the liver, increasing overall glucose production in the macroenvironment. (αMSH) α-Melanocyte-stimulating hormone, (Lcn2) Lipocalin 2, (NPY) neuropeptide Y, (MuRF1) muscle RING finger protein 1, (ATGL) adipose triglyceride lipase, (HSL) hormone-sensitive lipase.

Tumors impose profound and coordinated changes in host metabolism that extend from systemic to tissue- and organ-specific metabolism. Through dynamic tumor–host interactions, cancers rewire energy balance, redistribute nutrients, and disrupt metabolic homeostasis in distant organs, collectively shaping disease progression and therapeutic response (Fig. 1). One of the most striking clinical manifestations of this systemic metabolic disruption is cancer associated cachexia (CAC), a complex syndrome marked by involuntary weight loss, skeletal muscle wasting and depletion of adipose tissue. CAC profoundly compromises patient quality of life and treatment tolerance, and is estimated to directly contribute to death in 20%–30% of patients with advanced stage cancers (Argilés et al. 2014). At a physiological level, CAC is marked by severe dysregulation of whole-body energy metabolism. Patients frequently exhibit elevated resting energy expenditure despite a reduced appetite, together with widespread alterations in protein, lipid and carbohydrate metabolism (Petruzzelli and Wagner 2016). These intersecting metabolic abnormalities initiate a self-reinforcing cascade of organ dysfunction: progressive muscle wasting compromises respiratory mechanics and cardiac function, while sustained weight loss and hepatic metabolic exhaustion reduce resilience to therapy and increase treatment-induced toxicity. As a result, mortality in advanced cachexia often reflects progressive multiorgan failure within the host rather than direct tumor burden alone (Ferrara et al. 2022). Beyond its overt clinical consequences, CAC serves as a compelling example of the profound capacity of tumors to reprogram host metabolism at the organismal level, underscoring the importance of understanding the systemic metabolic consequences of cancer beyond the tumor itself.

### Glucose metabolism

Glucose intolerance represents one of the earliest recognized metabolic abnormalities in cancer patients and constitutes a major disruption of host energy homeostasis with profound consequences (Rohdenburg et al. 1919). Across different tumor types, systemic reprogramming of glucose metabolism, characterized by increased whole body glucose production (Kokal et al. 1983; Heber et al. 1985) and insulin resistance (Màrmol et al. 2023), is widely reported and is a part of the pathophysiology of CAC patients (Petruzzelli and Wagner 2016; Berriel Diaz et al. 2024). Elevated gluconeogenesis is a critical contributor to this phenotype, often driven by metabolic exchange between peripheral tissues and the liver (Holroyde and Reichard 1981; Berriel Diaz et al. 2024). Activation of the highly inefficient Cori cycle (Cori 1981) wherein lactate in circulation, derived from the tumor itself as well as peripheral tissues, is continuously converted to glucose, imposes an energy burden on the host while sustaining glucose availability for highly glycolytic tumors (Holroyde et al. 1975; Gold 1987; Friesen et al. 2015). A phenomenon commonly observed in cachectic cancer patients contributing to weight loss in the patient population (Holroyde et al. 1984). Hepatic gluconeogenesis in cancer can also be fueled by the glucose–alanine cycle (Cahill cycle), which mobilizes skeletal muscle-derived alanine to support hepatic gluconeogenesis (Felig et al. 1970; Arbeit et al. 1982). Mechanistically, tumor-induced alterations in hepatic glucose metabolism are mediated through both inflammatory and neurogenic pathways. In lung adenocarcinomas, elevated circulating cytokines, such as IL-6, activate hepatic STAT3–Soc3 signaling, disrupting circadian metabolic regulation and inducing a state of lower insulin sensitivity thereby leading to deregulated glucose production in the liver (Masri et al. 2016). In contrast, in nonmetastatic breast cancer models, aberrant hypothalamic neuronal activity rather than systemic inflammation was found to drive hepatic glucose dysregulation (Borniger et al. 2018). Together, these findings reveal how tumors exploit different regulatory circuits to reprogram host glucose metabolism to facilitate tumor growth by metabolically fueling cancer cells at the expense of the host energy reserves.

### Protein metabolism and skeletal muscle wasting

Systemic protein metabolism, another important pillar of the body's energy homeostasis is also profoundly disrupted in cancer, with increased whole-body protein turnover and catabolism documented in both preclinical models and patients (Norton et al. 1981a,b; Fearon et al. 1988). Patient studies also show that advanced disease had significantly higher rates of protein synthesis, turnover and catabolism (Carmichael et al. 1980; Borzotta et al. 1987), linking high protein metabolism to tumor progression. Plasma free amino acid (PFAA) constitutes a minor but metabolically active part of the total body amino acid pool (Lai et al. 2005). PFAA profile of patients with solid tumors reported a significant reduction of gluconeogenic amino acids like threonine, serine, glycine along with an increase in free tryptophan and glutamic acid in cases of lung cancer (Cascino et al. 1995). In other solid malignancies like breast cancer, significant increases in ornithine, glutamic acid and free tryptophan were observed (Cascino et al. 1995). Moreover, Georgiannos et al. (1995) showed that significant reductions in PFAA are observed in gastrointestinal cancer patients that have weight loss when compared to weight stable early-stage patients. A major source of amino acid redistribution in the host is the wasting of muscle mass, one of the main tissues and processes affected in CAC. At a molecular level, cancer-induced skeletal muscle wasting is chiefly a result of increased muscle proteolysis and has been shown to be induced by the activation of the ubiquitin–proteosome system (UPS) (Zhang et al. 2013; Sandri 2016). Activation of UPS in the skeletal muscle is proposed to be through the increased expression of atrophy-related genes muscle RING finger protein-1 (MuRF1) and muscle atrophy F-box (MAFbx) (Lecker et al. 2004; Egerman and Glass 2014), with the genetic ablation of *MuRF1* conferring resistance to muscle wasting in pancreatic cancer models (Neyroud et al. 2023). Autophagic degradation of proteins further contributes to muscle catabolism in both experimental models (Penna et al. 2013) and patients (Aversa et al. 2016). These catabolic programs are activated by tumor derived factors, including proteolysis inducing factor (PIF), and by host-derived inflammatory cytokines such as TNFα and IL-6 (Li and Reid 2000; Tisdale 2003; Haddad et al. 2005). Beyond driving muscle loss and consequently impairing quality of life, muscle wasting compromises treatment tolerance and exacerbates therapy-induced toxicity (Jensen et al. 2023). Collectively, tumor-induced activation of proteolytic and autophagic programs in skeletal muscle mobilizes systemic amino acid pools to fuel malignant growth (Luo et al. 2014; Porporato 2016; Pranzini et al. 2024), coupling cachexia directly to tumor metabolic demand.

### Lipid metabolism and adipose tissue reprogramming

A parallel collapse in the body's energy reserves in the form of adipose tissue wasting represents another defining feature of CAC, driven primarily by elevated lipolysis and adipose dysfunction (Bing et al. 2006; Petruzzelli and Wagner 2016; Babic et al. 2023; Berriel Diaz et al. 2024; Snoke et al. 2025). Increased activity of adipose triglyceride lipase (ATGL) and hormone-sensitive lipase (HSL) promotes fatty acid mobilization, with genetic disruption of these enzymes mitigating white adipose tissue (WAT) loss and systemic metabolic dysfunction in tumor-bearing mice (Agustsson et al. 2007; Das et al. 2011). Tumor- and host-derived cytokines, including the elevation of IL-6 and leukemia inhibitory factor (LIF), directly stimulate lipolysis (Arora et al. 2018 ; Pototschnig et al. 2023). IL-6 levels can also regulate WAT browning in CAC in lung and pancreatic tumors through the increased expression of uncoupling protein 1 (UCP1), thereby increasing resting energy expenditure and driving wasting (Petruzzelli et al. 2014). Tumor-derived parathyroid-hormone-related protein (PTHrP) similarly was shown to directly cause WAT browning and wasting in a model of lung adenocarcinoma (Kir et al. 2014)

Adipose tissue wasting and systemic lipid mobilization have profound secondary effects on hepatic metabolism as well, as metabolites released from adipose depots reshape the hepatic lipidome (Zhang et al. 2024b). Distant tumors can also directly alter lipid metabolism in the liver. Wang et al. (2023) show that lipid cargos from tumor-derived EVs, particularly palmitate, induce metabolic dysfunction in the liver and promote fatty liver disease. On the other hand, Li et al. (2024a) show that breast cancer-derived EV carrying miR-9-5p regulates cholesterol homeostasis in the liver by inducing the expression of 3-hydroxy-3-methylglutaryl-CoA reductase (HMGCR) and cholesterol 25-hydroxylase (CH25H), two crucial enzymes in the cholesterol synthesis pathway (Li et al. 2024a). Consistent with these findings, cancer patients exhibit widespread deregulation of systemic lipid profiles, including significant alterations in cholesterol, triglycerides and fatty acid levels (Wolrab et al. 2021; Lee et al. 2022). Lower levels of plasma cholesterol, most noticeably high-density lipoprotein cholesterol (HDL-C) has been reported in hematological malignancies (Kuliszkiewicz-Janus et al. 2008). On the other hand, cholesterol and low-density lipoprotein cholesterol (LDL-C) in gastric cancer patients showed lower levels in those with peritoneal metastasis whereas patients with lung metastasis showed an increase in LDL-C (Zhang et al. 2024a). Interestingly, increased low-density lipoprotein (LDL) levels in circulation of patients with metastatic tumors across cancer types correlate with metastatic burden and clinical outcome (Ghahremanfard et al. 2015). Moreover, the metabolic consequences of systemic lipid alterations in cancer patients are often dire comorbidities, which include cardiovascular events like heart failure, cardiomyopathy, and arrhythmia (Strongman et al. 2019). These findings position adipose tissue as a dynamic metabolic reservoir that tumors exploit through cytokine-driven lipolysis and thermogenic remodeling, reshaping systemic lipid availability and organ homeostasis, with profound consequences for tumor progression and prognosis.

Longitudinal studies indicate that the adipose tissue is affected early in the course of cancer associated cachexia with loss of fat mass often shown to emerge early during development of CAC, preceding overt skeletal muscle wasting (Das et al. 2011; Weber et al. 2022). However, as cachexia progresses, metabolic cross-talk between adipose tissue and skeletal muscle establishes a self-reinforcing catabolic cycle in which muscle wasting further accelerates adipose tissue lipolysis, amplifying systemic energy imbalance and tissue degeneration (Petruzzelli and Wagner 2016; Rupert et al. 2021). Importantly, this metabolic state is fundamentally distinct from physiological starvation. During starvation, depletion of glycogen stores triggers a coordinated adaptive transition toward fatty acid oxidation and ketone body production to preserve energy homeostasis (Anton et al. 2018). In contrast, CAC is characterized by simultaneous activation of multiple maladaptive catabolic pathways driven by tumor- and host-derived inflammatory mediators. Cytokines such as TNF-α and IL-6 exert pleiotropic effects across metabolic organs, promoting adipose tissue lipolysis, stimulating hepatic gluconeogenesis, and suppressing anabolic processes including protein, lipid, and glycogen synthesis (Ferrer et al. 2023; Iaia et al. 2025). Collectively, these observations support a model in which tumors induce a chronic, systemic catabolic state that progressively dismantles the body's energy reserves through coordinated dysfunction across adipose tissue, skeletal muscle, and the liver.

### Neuroendocrine regulation

The neuroendocrine system has emerged as a central regulatory axis through which tumors reshape systemic metabolism, energy balance, and behavior (Slominski et al. 2023). Rather than functioning solely as localized metabolic consumers, tumors actively engage bidirectional communication with the central nervous system to reprogram organismal physiology in ways that support disease progression. Across multiple non-CNS cancer models, transcriptomic and metabolomic analyses of the brain reveal widespread alterations in amino acid metabolism, protein synthesis, and sphingolipid pathways, together with extensive remodeling of hypothalamic signaling networks (Dwarkasing et al. 2015; Kovalchuk et al. 2018; Huisman et al. 2022). These findings suggest that tumors induce a systemic neuro–metabolic state in which central nutrient sensing and energy homeostasis become fundamentally dysregulated.

A major consequence of this rewiring is disruption of hypothalamic circuits that normally couple feeding behavior to energetic demand. Multiple tumor-induced signals converge on neuroendocrine pathways controlling appetite and energy expenditure. For example, expression of lipocalin-2 (Lcn2), an anorexigenic factor, is strongly induced within the hypothalamus of mice bearing pancreatic tumors (Huisman et al. 2022), while levels of the orexigenic neuropeptide Y (NPY) are reduced in cachectic rodents (Meguid et al. 2004). Although circulatory ghrelin levels are elevated in cachectic patients, this increase is widely interpreted as a compensatory response to ongoing wasting rather than an effective restoration of appetite (Shimizu et al. 2003; Garcia et al. 2005; Fujitsuka et al. 2011). Together, these observations indicate that tumors uncouple appetite regulation from the physiological needs of the host, driving a state in which energy intake fails to match escalating systemic demand. The hypothalamic melanocortin system, a key integrator of peripheral nutrient and hormonal signals (Kim et al. 2014), has also emerged as a key node in cancer-associated neuroendocrine dysregulation. Elevated circulatory levels of leukemia inhibitory factor (LIF), observed in CAC patients (Iseki et al. 1995; Vaes et al. 2020), stimulate hypothalamic release of α-melanocyte stimulating hormone (α-MSH), a melanocortin peptide that suppresses food intake while increasing energy expenditure (Grossberg et al. 2010). In parallel, hypothalamic hypocretin (orexin) signaling, which regulates appetite, arousal, and sleep-wake behavior, becomes disrupted in mice bearing nonmetastatic mammary tumors, resulting in aberrant neuronal activity, disordered feeding behavior, and sleep fragmentation (Borniger et al. 2018). Collectively, these studies support a model in which tumors broadly perturb central neuroendocrine circuitry to establish a chronic catabolic state characterized by reduced nutrient intake, elevated energy expenditure, and behavioral dysfunction.

Cancer-associated neuroendocrine remodeling extends beyond hypothalamic appetite regulation to involve systemic stress-response pathways. Clinical studies have reported dysregulation of the hypothalamic-pituitary-adrenal (HPA) axis in cancer patients (Kanter et al. 2024), with both cachectic patients and tumor-bearing mouse models exhibiting elevated circulating glucocorticoid levels (Knapp et al. 1991; Russell and Tisdale 2005; Martin et al. 2022). As master regulators of the physiological stress response, glucocorticoids exert broad catabolic effects that reinforce tumor-associated metabolic dysfunction, including stimulation of skeletal muscle proteolysis (Goldberg 1969), hepatic gluconeogenesis (Xu et al. 2021) and adipose tissue lipolysis (Xu et al. 2009). Importantly, neuroendocrine dysregulation also manifests through altered autonomic signaling. In murine models of CAC, inflammation-driven disruption of the brain-liver vagal axis impairs hepatic protein metabolism, contributing to reduced therapeutic responsiveness and shortened survival (Garrett et al. 2025). Taken together, these findings support a conceptual framework in which tumors hijack neuroendocrine and autonomic regulatory circuits to integrate tumor-derived inflammatory and metabolic signals into organism-wide physiological programs. Through persistent activation of central catabolic pathways, tumors rewire host behavior, endocrine signaling, and peripheral metabolism in a coordinated manner that amplifies systemic wasting and sustains tumor-supportive metabolic states.

## Host metabolic reprogramming as an enabling axis of tumor progression

Cancer progression is not solely dictated by tumor-intrinsic metabolic programs or local microenvironmental metabolic constraints but is also profoundly shaped by the systemic metabolic state of the host. Host physiology, including germline genetics, age and environmental influences such as diet and lifestyle heavily influence how the tumor and its surrounding microenvironment evolve over time (Altea-Manzano et al. 2025). A widely recognized example of this interplay is obesity, where adipose tissue dysfunction and chronic metabolic inflammation create systemic conditions that promote cancer initiation and progression (Quail and Dannenberg 2019). More broadly, dietary composition plays a central role in shaping systemic metabolism and thereby influencing tumor behavior. High-fat diets, for instance, remodel circulating lipid availability in ways that can be exploited by metastasizing cancer cells to fuel disease progression (Altea-Manzano et al. 2023). Likewise, fructose supplementation, despite not being directly utilized by many tumor cells, is metabolized in the liver into circulating lipid species that subsequently support tumor growth and metabolic activity (Fowle-Grider et al. 2024).

Importantly, systemic metabolism can also exert tumor-suppressive effects. Decades of research on calorie restriction (CR) have demonstrated that reduced nutrient availability can suppress carcinogenesis and tumor progression (Hursting et al. 2010). Similarly, ketogenic diets, which elevate circulating ketone bodies, have been shown in some contexts to suppress glycolytic flux and impair tumor proliferation (Qin et al. 2024), although these responses appear highly dependent on tumor lineage and metabolic plasticity (Giuliani and Longo 2024). Together, these observations highlight that systemic metabolism is not merely a passive backdrop to cancer progression, but rather a dynamic regulator capable of either constraining or supporting tumor growth depending on physiological context.

**Figure 2.**
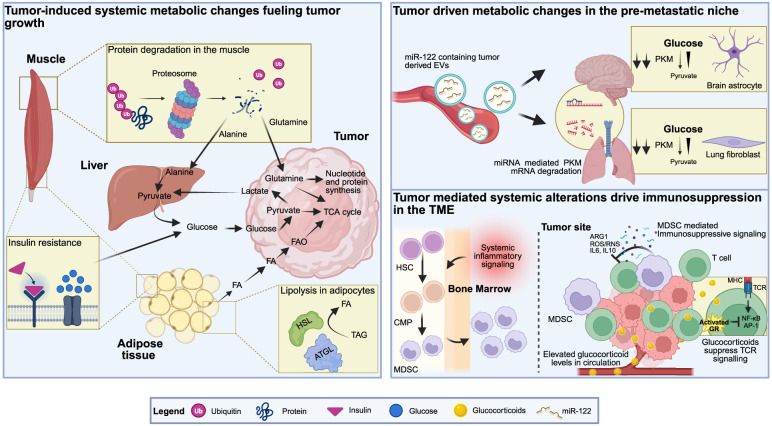
Tumor-induced changes in the macroenvironment propel cancer progression. (*Left*) Tumor-mediated rewiring of host metabolic pathways and redistribution of resources fuels energy metabolism in cancer cells. Skeletal muscle proteolysis provides glutamine, which fuels the TCA cycle and supports nucleotide and protein synthesis in tumor cells. Muscle-derived alanine is taken up by the liver and used for gluconeogenesis, generating glucose that is subsequently utilized by tumors to sustain glycolysis. Lactate produced by tumor glycolysis is returned to the liver, where it further drives gluconeogenesis, establishing a positive feedback loop. In parallel, pyruvate produced during glycolysis and fatty acids derived from adipose tissue lipolysis are also contributors to TCA cycle activity in tumor cells. Additionally, systemic insulin resistance elevates circulating glucose levels, further enhancing glucose availability and glycolytic flux within tumors. (*Top right*) Tumors hijack glucose metabolism in the distant site aiding the establishment of the premetastatic niche. miR-122 containing EVs shed by tumor cells posttranscriptionally regulate the levels of the rate-limiting glycolytic enzyme PKM in brain astrocytes and lung fibroblasts modulating glucose availability in the premetastatic niche. (*Bottom right*) Tumor-induced dysregulation of host inflammatory signaling disrupts the differentiation of immune populations in the bone marrow leading to the accumulation of MDSCs that mediate immunosuppressive signaling at the tumor site. Moreover, elevated host glucocorticoid levels can suppress TCR signaling in the tumor microenvironment furthering an immunosuppressive niche. (FA) Fatty acids, (FAO) fatty acid oxidation, (TAG) triglycerides, (ATGL) adipose triglyceride lipase, (HSL) hormone-sensitive lipase, (PKM) pyruvate kinase, (EV) extracellular vesicle, (HSC) hematopoietic stem cell, (CMP) common myeloid progenitor, (MDSC) myeloid-derived suppressor cells, (ARG1) arginase 1, (ROS/RNS) reactive oxygen species/reactive nitrogen species, (GR) glucocorticoid receptor.

While the systemic metabolic remodeling discussed previously is often viewed through the lens of its detrimental effects on the host, these alterations also generate conditions that can directly benefit the tumor. Growing evidence indicates that tumors actively reprogram host metabolism to establish a permissive systemic macroenvironment that enhances nutrient availability, suppresses antitumor immunity, and buffers cancer cells against environmental and therapeutic stress. Through coordinated metabolic rewiring of distant organs and tissues, tumors transform the host into an integrated metabolic network that supports tumor growth, metastatic dissemination, and persistence during therapy (Fig. 2).

### Dysregulated systemic host metabolism acts as an energy source fueling tumor growth

One of the earliest conceptual frameworks linking metabolism to cancer was articulated by Otto Warburg, who proposed that cancer cells preferentially engage aerobic glycolysis and in turn rapidly consume glucose to not only to meet their energetic demands but also to enable the synthesis of critical building blocks for the growing tumor population, a phenomenon later termed as the Warburg effect (Warburg et al. 1927). Subsequent work firmly established that metabolic competition within the TME is a defining feature of tumor progression (Lyssiotis and Kimmelman 2017). Cancer cells aggressively compete for glucose, often outcompeting infiltrating T cells and thereby suppressing T-cell glycolysis, a metabolic program critical for T-cell cytotoxicity (Chang et al. 2015). Beyond direct competition, cancer cells actively reprogram neighboring stromal cells to create metabolically favorable niches. Cancer-associated fibroblasts (CAFs) undergo aerobic glycolysis and export catabolized nutrients to fuel tumor metabolism, enriching the TME with energy substrates (Pavlides et al. 2009). Among these, lactate, traditionally viewed as a metabolic waste product of glycolysis, can be actively imported and utilized by cancer cells to sustain lactate-driven oxidative respiration (Sonveaux et al. 2008). Similarly, lipolysis in bone marrow adipocytes support leukemic cells through fatty acid transfer in models of acute myeloid leukemia (AML) (Shafat et al. 2017), while ovarian cancer cells metastasizing to the omentum exploit the lipid-rich adipose environment by inducing lipolysis and scavenging released fatty acids (Nieman et al. 2011). Amino acid availability within the TME further constraints tumor growth. Glutamine, in particular, serves as a critical carbon and nitrogen source, prompting cancer cells to upregulate amino acid transporters on their cell surface to sustain uptake under nutrient stress (Saito and Soga 2021). In parallel, CAFs in the TME induce glutamine synthetase (GS) expression to locally feed the glutamine dependencies of the adjacent cancer cells, reinforcing metabolic reprogramming within the microenvironment (Yang et al. 2016). Beyond glutamine, stromal cells can provide alternative amino acids that sustain tumor metabolism when canonical nutrients are limiting. In pancreatic cancer, pancreatic stellate cells (PSCs) undergo autophagy-dependent amino acid release and secrete alanine, which is taken up by cancer cells and used as a carbon source to support central carbon metabolism and biosynthetic needs under nutrient limitation (Sousa et al. 2016). Ammonia, a nitrogen rich byproduct derived from glutamine catabolism is another waste-derived metabolite that can be repurposed by tumor cells. Ammonia can be recycled by neighboring tumor cells for nonessential amino acid biosynthesis (Spinelli et al. 2017). Consistent with this broader model of stromal nutrient provisioning, CAF-derived EVs containing lipids, amino acids and metabolic intermediates can be internalized by cancer cells and utilized as alternative nutrient sources under conditions of nutritional stress (Zhao et al. 2016). Together, these studies illustrate how cancer cells co-opt surrounding nonmalignant cells to buffer metabolic stress and sustain growth within the confines of the TME.

Despite the identification of multiple metabolic dependences within the TME, therapeutic strategies targeting TME-restricted metabolic vulnerabilities has not lived up to its potential in the clinic (Long et al. 2019; Tannir et al. 2022). A central reason for this shortfall is the extraordinary metabolic plasticity of cancer cells and their capacity to extend metabolic control beyond the physical limitations of the tumor niche. While metabolic buffering within the TME enables tumors to survive local nutrient limitation, tumors layer systemic metabolic orchestration onto these local interactions, actively regulating host-wide nutrient fluxes to stabilize tumor metabolism and buffer metabolic stress at the organismal scale (Elia and Haigis 2021; Altea-Manzano et al. 2025). Early studies of tumor–host metabolic interactions revealed that tumors hijack systemic glucose metabolism, with the liver acting as a central metabolic hub. As alluded to previously, tumor-driven interorgan metabolic circuits sustain elevated systemic glucose availability through processes such as hepatic gluconeogenesis fueled by skeletal muscle-derived substrates including alanine, albeit at a substantial energetic cost to the host (Holroyde et al. 1975; Arbeit et al. 1982; Gold 1987). Consistent with the functional importance of these circuits, pharmacological inhibition of glycolysis with 2-deoxy-D-glucose (2-DG) significantly ameliorates muscle wasting and restricts tumor growth in a model of colorectal cancer (Wei et al. 2022). Mechanistic insight into tumor-driven systemic glucose dysregulation has further revealed direct tumor control over endocrine function. In breast cancer, EVs encapsulating miR-122 suppress insulin secretion from pancreatic β cells, inducing systemic hyperglycemia through impaired insulin exocytosis (Cao et al. 2022). Restoration of glucose homeostasis via insulin supplementation or pharmacologic glucose lowering using sodium–glucose cotransporter-2 (SGLT2) inhibitors significantly impairs tumor growth in triple-negative breast cancer models (Cao et al. 2022). Similar hijacking of host glucose metabolism has also been shown in leukemias, where the presence of leukemic cells induce insulin resistance across multiple organs tissue to elevate glucose levels, and where pharmacological redirecting of systemic glucose flux markedly restricts leukemia progression (Ye et al. 2018). Conversely, physiological redistribution of glucose toward the skeletal muscle through voluntary exercise slows tumor growth in rodents, underscoring the plasticity and therapeutic tractability of systemic metabolic allocation (Leitner et al. 2025). Importantly, the systemic metabolic influence of tumors extends well beyond glucose metabolism to encompass amino acid and nucleotide homeostasis, thereby reinforcing metabolic dependencies within the tumor microenvironment. Tumor burden drives coordinated changes in whole-body amino acid metabolism, reshaping circulating amino acid pools in ways that both support tumor growth and influence antitumor immune responses (Parker et al. 2020). These systemic adaptations function in concert with local stromal nutrient exchange within the TME, providing tumors with a continuous metabolic supply even under conditions of fluctuating local nutrient availability. For example, cancer-associated fibroblasts (CAFs) and tumor-associated macrophages (TAMs) serve as important local sources of nucleosides that sustain nucleotide metabolism within tumors (Halbrook et al. 2019; Yuan et al. 2022). At the same time, peripheral tissues contribute metabolites such as glutamine that can be utilized by tumors to support de novo nucleotide synthesis (Luo et al. 2014). Beyond local production, tumors also exploit systemic salvage pathways, as circulating nucleotide precursors can be recycled across tissues to maintain intracellular nucleotide pools and support continued proliferation (Tran et al. 2024). Tumors also transcriptionally rewire the distant metabolic organs to access additional nutrient reservoirs. A good example of this is the depletion of master metabolic regulator HNF4α in the liver of breast and pancreatic cancer-bearing mice, which disrupts urea cycle function and nitrogen metabolism, reshaping systemic nitrogen availability to favor tumor growth and disease progression (Goldman et al. 2023), a phenomenon also observed in models of cancer cachexia (Garrett et al. 2025). Skeletal muscle similarly serves as a systemic nutrient source for cancer cells, with MuRF1-driven muscle catabolism fueling pancreatic cancer growth (Neyroud et al. 2023) and muscle-derived glutamine directly incorporated into colorectal cancer metabolism (Luo et al. 2014). Similarly, increased lipolysis in adipose tissue increases circulating lipid availability, supporting rapid growth of nonglycolytic tumors that rely on fatty acid oxidation (Liu 2006; Huang et al. 2018). Additional nutrient sources may also arise through tumor-associated remodeling of the gut microbiome, as observed in colorectal cancer patients, where altered microbial composition and metabolic activity drive the accumulation of metabolites such as lactate and polyamines that can be directly utilized to fuel tumor growth (Arruabarrena-Aristorena et al. 2018; Bautista et al. 2026). Together, these findings demonstrate that local metabolic interactions within the TME are necessary but insufficient to sustain malignant growth. Instead, tumors integrate local buffering with systemic metabolic orchestration, converting host organs into a distributed resource network that fine-tunes tumor metabolism, buffers therapeutic stress, and enables disease progression at the organismal scale.

### Tumor-mediated systemic immunomodulation as an instructional driver of tumor growth

The relationship between tumors and host immunity has long been recognized as a central determinant of cancer progression, dating back to the pioneering observations of William Coley (1891, 1991), which first hinted at a profound connection between immune activation and tumor fate. Over the past century, this relationship has evolved from being viewed as a passive host response to malignancy into a dynamic and actively co-opted axis through which tumors instruct and remodel systemic immunity to support their own survival and dissemination (Mantovani et al. 2008; Hiam-Galvez et al. 2021). Tumors not only evade immune destruction locally within the TME, but also exert organism-wide control over immune cell differentiation, inflammatory tone, and metabolic fitness, thereby establishing a systemic immune landscape permissive to malignant progression.

A defining feature of this systemic immune remodeling is chronic inflammation and has long been put forward as a major regulator of cancer, as reviewed extensively elsewhere (Mantovani et al. 2008; Grivennikov et al. 2010; Greten and Grivennikov 2019). Here we focus on how inflammatory signals in the tumor bearing host modulate host physiology and metabolism to favor cancerous disease progression.

As the tumor progresses, elevated levels of inflammatory mediators such as TNF-α and IL-6 do far more than simply reflect disease burden; rather, they function as active instructive signals that reinforce malignant growth. Within the TME, these cytokines promote tumor cell survival and proliferation while stimulating angiogenesis and recruitment of protumorigenic myeloid populations (Giri et al. 2001; Grivennikov et al. 2009; Grivennikov et al. 2010). Importantly, these inflammatory programs extend beyond the local tumor niche to reshape immune surveillance and metabolic homeostasis across the host, coupling systemic inflammation to broader physiological dysfunction (Hiam-Galvez et al. 2021). This inflammatory remodeling is deeply intertwined with the systemic metabolic reprogramming discussed in previous sections. Cytokines such as TNF-α and IL-6 act as central mediators linking immune dysregulation to host catabolism, driving skeletal muscle proteolysis and adipose tissue lipolysis to mobilize nutrients that ultimately support tumor growth (Ferrer et al. 2023; Iaia et al. 2025). In parallel, systemic IL-6 promotes hepatic insulin resistance and altered glucose availability for highly glycolytic tumors while reinforcing a proinflammatory hepatic state (Klover et al. 2003; Cai et al. 2005). Through these interconnected pathways, inflammation and metabolic dysfunction form a self-reinforcing systemic circuit that sustains both tumor progression and host wasting.

Systemic immune signaling can also exert direct control over metastatic behavior. Chronic inflammatory signaling within distant organs has been shown to govern the reawakening of dormant disseminated cancer cells (DCCs). In the lung, inflammation-induced neutrophil extracellular trap (NET) formation remodels the local microenvironment and triggers dormant breast cancer cells to re-enter the cell cycle and form overt metastatic lesions (Albrengues et al. 2018). These findings demonstrate that organism-wide immune activation can directly dictate cell-intrinsic tumor fate at distant metastatic sites. In parallel, tumors profoundly reshape hematopoietic output to bias systemic immunity toward immunosuppressive and tumor-supportive states. Tumor-bearing hosts exhibit expansion of neutrophils, eosinophils, and monocytes together with depletion of dendritic cells, B cells, and T cells in peripheral compartments (Allen et al. 2020 ; Hiam-Galvez et al. 2021). Importantly, these alterations are not merely correlative; systemic immune dysfunction can be reversed following tumor removal or cytokine blockade, highlighting a causal role for tumor-derived inflammatory signals in remodeling hematopoiesis (Allen et al. 2020). Expansion of immature myeloid populations further amplifies local immune suppression upon recruitment into the TME, where they inhibit adaptive immune surveillance and reinforce tumor-permissive conditions (Gabrilovich 2017).

Beyond broad hematopoietic bias, tumors deploy highly specific mechanisms to suppress immune competence across both peripheral tissues and the TME. Tumor-derived G-CSF suppresses IRF8 expression in dendritic cell progenitors, selectively impairing the development of type 1 conventional dendritic cells (cDC1s), which are critical for antitumor T-cell priming (Meyer et al. 2018). Similarly, tumor-derived VEGF inhibits dendritic cell maturation and antigen-presenting capacity (Gabrilovich et al. 1996). Tumors also propagate immune suppression systemically through EVs. In metastatic melanoma, EV-associated PD-L1 suppresses peripheral T-cell activation, effectively extending immune checkpoint-mediated exhaustion beyond the confines of the TME and promoting organism-wide immune suppression (Chen et al. 2018).

Importantly, tumors also suppress immunity by reshaping the systemic nutrient landscape required for immune cell function. Inflammation-driven activation of indoleamine 2,3-dioxygenase (IDO) reduces circulating tryptophan levels while increasing kynurenine accumulation, creating a metabolically immunosuppressive environment that constrains effector T-cell proliferation and cytotoxicity (Altea-Manzano et al. 2025). These effects can be further amplified by tumor-associated alterations in the gut microbiome, such as enrichment of *Fusobacterium nucleatum*, which promotes IDO1 expression in host immune cells and reinforces systemic immune tolerance (van Baren and Van den Eynde 2015). Likewise, elevated arginase activity detected in the peripheral blood of gastric cancer patients depletes circulating arginine, impairing T-cell effector function and cytotoxic responses (Wu et al. 1994; Munder 2009; Geiger et al. 2016). Collectively, these observations reveal that tumors regulate antitumor immunity not only through checkpoint signaling, but also through systemic control of the metabolic substrates necessary for immune competence.

Superimposed on these mechanisms, circulating metabolites themselves can directly reprogram immune cell state, providing an additional layer of systemic immune instruction. Tumor-associated gut dysbiosis elevates secondary bile acids such as deoxycholic acid (DCA), which promote immunosuppressive macrophage polarization and impair CD8^+^ T-cell function (Cong et al. 2024; Liu et al. 2025; Varanasi et al. 2025). Similarly, elevated circulating MMA, an age-associated metabolite linked to perturbed propionate metabolism and vitamin B12 status (Tejero et al. 2024), drives transcriptional and functional exhaustion of CD8^+^ T cells, including induction of the exhaustion regulator TOX and impaired effector programs (Tejero et al. 2025). Mechanistically, MMA-induced dysfunction is associated with suppression of NADH-regenerating reactions within the TCA cycle and broader defects in mitochondrial function (Tejero et al. 2025), demonstrating that systemic metabolic alterations can erode antitumor immunity even in the absence of direct local tumor cues.

Together, these findings support a model in which tumors transform systemic immunity into an actively instructed, metabolically constrained, and chronically inflammatory state that favors tumor persistence and progression. Through coordinated control of immune differentiation, inflammatory signaling, nutrient availability, and immune cell metabolic fitness, tumors extend immune regulation far beyond the local TME, effectively converting host immunity from a barrier to malignancy into a systemic enabler of disease progression.

### Hijacking the neuronal circuits to further disease progression

The nervous system constitutes a powerful regulatory axis through which tumors exert control over host metabolism, immunity and stress responses (Amit et al. 2025). The hypothalamus, as a central integrator of peripheral cues governing energy balance and endocrine output, is particularly well positioned to mediate tumor–host communication (Kim et al. 2014). Early studies established that the central nervous system (CNS) modulates the growth of extracranial tumors (Cao et al. 2010; Pan et al. 2021), but more recent work reveals a bidirectional interaction in which tumors actively engage neural circuits to promote disease progression.

One mode of neuronal engagement operates through central neuroendocrine control of systemic immunity. Extracranial tumors have been shown to recruit the hypothalamic-pituitary circuit to secrete α-MSH, in turn promoting tumor progression by enhancing myelopoiesis in bone marrow progenitor cells (Xu et al. 2022). Similarly, activation of catecholaminergic (CA) neurons in the ventrolateral medulla (VLM) drives tumor progression by suppressing CD8^+^ T-cell-mediated immunity (Zhang et al. 2021). Dysregulation of the HPA axis further amplifies these effects, as elevated glucocorticoid levels, a common occurrence in cancer patients (Flint et al. 2016), are known to impair T-cell activation and effector function (Cain and Cidlowski 2017). Together, these studies establish that tumors can leverage central neural and endocrine pathways to systemically modulate immune tone and create conditions permissive for malignant progression. Distinct from these immune-centric neuroendocrine effects, emerging work demonstrates that tumors also directly engage neural circuits through tumor-derived signals and reciprocal metabolic interactions, enabling cancer cells to adapt to environmental stress independent of immune modulation. In this context, tumor-derived cytokines, such as LIF and galectin-3 (GAL3), increase phosphorylated ribosomal S6, a marker of neuronal activity, in the paraventricular nucleus of the hypothalamus (PVN) and other brain regions (Xu et al. 2024). Importantly, blockage of these tumor cell-derived cytokines abolished the tumor-induced neuronal activation and restricts tumor progression (Xu et al. 2024).

Beyond central circuits, tumors also exploit the peripheral nervous system (PNS) to withstand metabolic stress imposed by the TME. Tumors in nutrient-poor environments stimulate nociceptive neurons to release calcitonin gene-related peptide (CGRP), which induces cytoprotective autophagy in cancer cells by disrupting mTORC1 signaling and thereby enhances cancer survival (Zhang et al. 2022). Similarly, pancreatic cancer cells have been shown to rely on peripheral neurons as a source for exogenous serine under nutrient stress, directly linking neural inputs to tumor metabolic resilience (Banh et al. 2020). These findings support the idea that neural circuits can serve as conditional metabolic support systems, buffering tumors against local nutrient scarcity. This capacity for neural co-option is most pronounced in tumors growing within, or metastasizing to, the CNS. Venkatesh et al. (2019) demonstrated that gliomas form functional synapses with neurons and electrically integrate into the neuronal circuits. Targeting these electrochemical communications between neuron-glioma synapses reduced glioma growth and prolonged survival in tumor models (Venkatesh et al. 2019). Notably, synapse-like tumor–neuron interactions are not confined to the brain. In small cell lung cancer (SCLC), presynaptic vagal fibers expressing VGluT1 colocalize with postsynaptic HOMER1 in tumor cells, suggesting functional neuron–cancer synapses in peripheral tissue (Sakthivelu et al. 2025). Cancer cells metastasizing to the brain have also been shown to take advantage of these synapse-like structures where they physically interact with neurons to promote tumor growth. Breast cancer cells that metastasize to the brain form pseudotripartite synapses with glutamatergic neurons, activating N-methyl-D-aspartate receptors (NMDARs) in cancer cells, which is required for metastatic outgrowth (Zeng et al. 2019). Synaptic integration was also found to be of prominence in SCLCs where cancer cell-neuron synapses promoted invasion and growth of cancer cells in the intracranial space (Savchuk et al. 2025). Astrocytes further amplify neural–tumor metabolic coupling within the brain microenvironment. Lung and breast cancer cells form carcinoma–astrocyte gap junctions that activate STAT1 and NF-κB signaling, promoting tumor growth and therapy resistance (Chen et al. 2016). In parallel, tumor-derived EVs carrying miR-199b-5p disrupt the metabolic coupling between neurons and astrocytes, enriching the brain microenvironment with extracellular metabolites that fuel the growth of metastatic breast cancer cells (Ruan et al. 2024). Collectively, these studies position both central and peripheral neural circuits not as passive bystanders but as actively co-opted regulators that integrate immune suppression, metabolic support, and stress adaptation across local and systemic scales to drive cancer progression.

### Tumor-induced systemic metabolic education of the premetastatic niche

The establishment of distant metastasis remains the major cause of cancer-related mortality and often leads to worse outcomes after primary treatment (Gerstberger et al. 2023). Increasing evidence indicates that successful metastatic colonization is not determined solely by the intrinsic properties of disseminated cancer cells (DCCs), but also by the establishment of a permissive premetastatic niche (PMN) within distant organs prior to tumor cell arrival. The formation of these distally conditioned microenvironments is driven by tumor-induced systemic metabolic, immune, and neuroendocrine alterations that remodel distant tissues into permissive ecosystems capable of supporting DCC survival and metastatic outgrowth (Peinado et al. 2017). A central mechanism underlying PMN formation is the tumor-driven release of secreted factors, including cytokines, growth factors and EVs, that instruct stromal, immune and metabolic programs in future metastatic sites (McAllister and Weinberg 2014; Alečković et al. 2019). A clear example of metabolic conditioning comes from breast cancer-derived EVs carrying miR-122, which suppress glucose uptake in distant organs like the brain and lung by downregulating glycolysis through suppression of the glycolytic enzyme pyruvate kinase (PKM) in the nonmalignant tissue, conferring a growth advantage to the DCCs by making glucose available to them (Fong et al. 2015). In parallel, systemic metabolites can function as “endocrine-like” instructional cues that reprogram stromal states relevant to metastatic progression. Notably, methylmalonic acid (MMA), an age-induced metabolite (Tejero et al. 2024) that can also be produced by aggressive tumor cells (Gomes et al. 2022), drives activation of fibroblasts, promoting acquisition of CAF features (Li et al. 2022). Mechanistically, MMA induces reactive oxygen species (ROS) and activates TGFβ signaling, remodeling CAF programs to enhance cancer progression, metastasis, and drug resistance (Li et al. 2022). PMN formation can also proceed through indirect systemic relay mechanisms, whereby tumor-driven signaling cascades reprogram intermediary tissues that then propagate prometastatic signals. In pancreatic cancer, cancer cells trigger the release of IL-6 from fibroblasts in the TME, which in turn signals distally to activate STAT3 in hepatocytes. These STAT3-activated hepatocytes recruit immunosuppressive myeloid cells and activate hepatic stellate cells that remodel the extracellular matrix, subsequently making the liver environment susceptible to metastasis (Lee et al. 2019). This illustrates how local tumor–stromal interactions are amplified through systemic inflammatory and stromal circuits to condition distant organs.

Beyond stromal education, systemic metabolic cues can directly prime tumor cells toward metastatic competence. In aged hosts, MMA is transported via extracellular vesicles to tumor cells, where it induces transcriptional programs associated with EMT-like plasticity, invasiveness, and therapy resistance (Gomes et al. 2020). These host-derived EV metabolic signals can therefore cooperate with PMN remodeling by lowering intrinsic tumor-cell barriers to dissemination and outgrowth, effectively aligning tumor cell state with a permissive systemic environment. Tumors may further shape PMNs by reprogramming circulating and bone marrow–derived cell populations that later act at metastatic sites. Certain breast cancers release osteopontin (OPN) into circulation, altering bone marrow cell states in the host and promoting protumorigenic programs that support lung metastasis (McAllister et al. 2008). Similarly, in melanoma, tumor-derived exosomes carrying the receptor tyrosine kinase MET educate bone marrow progenitors toward a provasculogenic phenotype, critically regulating metastatic tumor burden at distant sites (Peinado et al. 2012). Moreover, endocrine alterations also contribute to systemic conditioning, as elevated glucocorticoid levels observed in cancer patients can potentiate breast cancer dissemination and metastatic colonization (Obradović et al. 2019). Collectively, these findings define PMN formation as a layered, systemically orchestrated process in which tumors integrate metabolic, inflammatory, and EV-mediated signaling to synchronously precondition both distant tissues and tumor cell state for metastatic progression.

### Therapy resistance driven by macroenvironmental reprogramming

Resistance to anticancer therapies is increasingly recognized as an emergent property of tumor–host interactions that extend beyond cancer cell–intrinsic mechanisms and the local TME. While neighboring immune cells (Li et al. 2024b) and fibroblasts (Masuda 2025) within the TME are well-established contributors to therapeutic resistance, the host macroenvironment and tumor-induced reprogramming of the host macroenvironment imposes additional, systemic constraints that shape treatment efficacy across multiple therapeutic modalities.

A major axis through which the macroenvironment influences therapy response is systemic immune remodeling, which strongly influences the efficacy of immunotherapies, particularly in advanced disease. Expansion of myeloid derived suppressor cells (MDSCs) has been shown to blunt antitumor immune responses and reduce the efficacy of immunotherapies (Lasser et al. 2024). Accordingly, the frequency of MDSCs have been shown to correlate with the clinical response of melanoma patients treated to the CTLA-4 inhibitor ipilimumab, with a lower frequency of monocytic MDSCs predicting better patient response (Meyer et al. 2014), underscoring how systemic immune bias can predetermine immunotherapy outcomes.

Superimposed on immune remodeling, endocrine stress responses impose additional constraints on therapy response by acting independently on immune competence. Consistent with this, heightened glucocorticoid signaling has been shown to blunt the efficacy of immune checkpoint inhibitors, reducing antitumor immune responses and limiting clinical benefit in patients receiving immunotherapy (Deng et al. 2021; Polyakov et al. 2025). In this context, endocrine dysregulation functions as a systemic immune checkpoint that constrains immunotherapy effectiveness independently of tumor-intrinsic resistance mechanisms. In parallel, glucocorticoid signaling directly reprograms tumor cell fate, promoting phenotypic plasticity that enables escape from targeted therapies. In lung cancer, activation of the glucocorticoid receptor induces a drug-tolerant dormant state characterized by reduced proliferative signaling and broad resistance to multiple targeted agents (Prekovic et al. 2021). This hormonally driven shift in tumor cell state underscores how systemic stress signals can stabilize nonproliferative, therapy-refractory phenotypes that persist. These endocrine adaptations intersect with broader inflammatory and metabolic programs that further condition therapeutic sensitivity. Elevated inflammatory cytokines are established modulators of treatment efficacy (Briukhovetska et al. 2021), and therapeutic targeting of inflammatory pathways can restore drug responsiveness. Accordingly, in ovarian cancer models, blockade of interleukin-6 using the humanized antibody siltuximab significantly improves response to chemotherapy, illustrating how dampening systemic inflammation can recondition the host environment to support chemotherapy efficacy (Stone et al. 2012). Chronic inflammation also drives systemic insulin dysregulation, with direct consequences for targeted therapies. In line with this, hyperinsulinemia potentiates resistance to PI3K inhibitors in solid tumors (Hopkins et al. 2018; Noch et al. 2023) and has been linked to resistance to anti-EGFR therapies (Chiu et al. 2020) Similarly, hyperuricemia, often a complication of hematological cancers (Kunlayawutipong et al. 2024) can modulate sensitivity to antimetabolite chemotherapy (Cantor et al. 2017). These studies highlight how the host metabolic state can override tumor-intrinsic drug sensitivities by reshaping systemic signaling landscapes.

A final, often underappreciated dimension of tumor–host cross-talk in therapy resistance lies in the tumor's ability to reprogram host drug metabolism, thereby reshaping pharmacokinetics, toxicity, and therapeutic windows at the organismal level. In line with this, extrahepatic malignancies suppress expression of hepatic CYP3A enzymes, key regulators of drug metabolism (Zanger and Schwab 2013), likely through inflammation-mediated signaling (Charles et al. 2006; Kacevska et al. 2011). Reduced CYP3A expression narrows therapeutic windows and increases toxicity risk, thereby limiting dose intensity and treatment durability in cancer patients (Lee et al. 2023).

Taken together, these observations redefine therapy resistance as a host-conditioned phenotype that emerges from coordinated immune, endocrine, inflammatory–metabolic, and pharmacologic reprogramming. Rather than arising solely from genetic or epigenetic changes within cancer cells, resistance is actively shaped by tumor-driven alterations in the systemic macroenvironment that constrain therapeutic efficacy even in the presence of potent tumor-targeted agents.

## Cancer survivorship and long-term systemic consequences of tumor–host reprogramming

Advances in early detection, targeted therapies, and supportive care have dramatically increased the number of individuals living years or even decades after a cancer diagnosis (Siegel et al. 2024). As survival improves, however, a new clinical and biological reality has emerged: cancer survivorship. Broadly defined, survivorship encompasses individuals with active disease, patients undergoing therapy, and those who have completed treatment, all of whom may experience persistent alterations in host physiology that extend far beyond the tumor itself. Increasing evidence suggests that the systemic reprogramming imposed by cancer and its treatment does not fully resolve following tumor eradication. Rather, tumor growth and anticancer therapies leave durable imprints across metabolic, immune, neuroendocrine, and organ systems that can persist long after the primary disease is clinically controlled.

This emerging view reframes survivorship not as a static postcancer state, but as a biological continuum shaped by prior tumor–host interactions and treatment exposure. During active disease, tumors establish widespread systemic programs that remodel metabolism, immunity, and tissue function to support malignant progression. Importantly, many of these alterations may persist or evolve after therapy completion, generating a stabilized yet biologically altered host state in which prior cancer-associated reprogramming continues to influence aging trajectories, organ homeostasis, and disease susceptibility. In this framework, survivorship represents the long-term physiological legacy of tumor–host coevolution rather than simply the absence of detectable malignancy.

**Figure 3.**
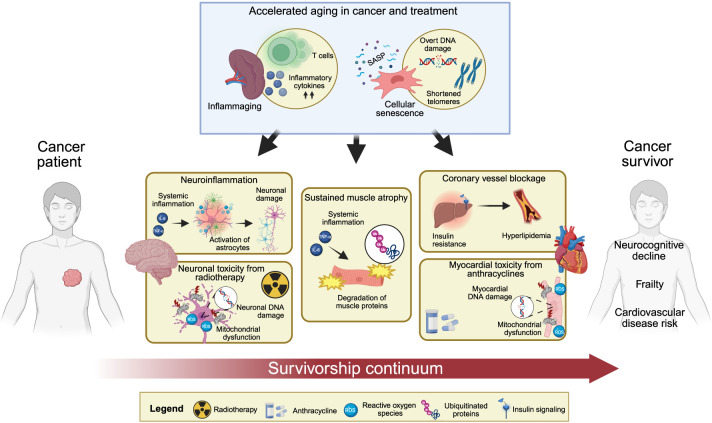
The survivorship continuum in cancer patients. Tumor and associated treatment modalities accelerate biological aging within the host, wherein an inflammaging-like proinflammatory phenotype and the induction of cellular senescence across host tissues shape the metabolic landscape of cancer survivors, increasing disease risk and impacting quality of life. In the central nervous system, treatment modalities like radiotherapy can affect neurons directly through an interplay of direct neuronal DNA damage, mitochondrial dysfunction and excessive ROS generation, while unresolved systemic inflammation causes hyperactivity in brain astrocytes furthering persistent neuroinflammation and causing damage to neuronal health. In the skeletal muscle, inflammatory signaling leads to sustained muscle atrophy through degradation of muscle proteins. In the cardiovascular system, metabolic alterations like insulin resistance contribute to hyperlipidemia, which consequently promotes coronary vessel blockage, while treatment with chemotherapies like anthracyclines are direct causes of myocardial toxicity via severe mitochondrial impairment, ROS generation and DNA damage in cardiomyocytes. Over the survivorship continuum, these cumulative effects manifest as neurocognitive decline, frailty and cardiovascular disease risk in cancer survivors. (SASP) Senescence-associated secretory phenotype, (TNF-α) tumor necrosis factor-α, (IL-6) interleukin-6.

Consistent with this concept, cancer survivors exhibit increased incidence of chronic metabolic, cardiovascular, neurological, and inflammatory disorders together with elevated all-cause mortality compared to cancer-naive populations (Wang et al. 2025). These observations suggest that cancer leaves a persistent systems-level imprint on the host, raising the possibility that many late effects of cancer and its treatment arise from durable rewiring of organismal physiology initiated during tumor progression itself (Fig. 3).

### Accelerated aging in cancer survivors

A growing body of evidence indicates that cancer history accelerates biological aging. Survivors exhibit long-term impairments in both physical and neurocognitive function (Siddique et al. 2021; Cespedes Feliciano et al. 2023), and molecular measurements using epigenetic clocks and clinical biomarkers corroborate increased biological age relative to chronological age when compared to cancer free controls (Wang et al. 2025). Mechanistic studies suggest these changes are not merely secondary to treatment toxicity but arise from tumor-driven reprogramming of host tissues. In lymphoma-bearing mice, tumor exposure alone induces premature aging signatures in young hosts with T cells isolated from tumor-exposed young mice adopting a transcriptional and epigenetic profile characteristic of aged T cells, accompanied by increased inflammatory cytokines and elevated senescence markers in peripheral tissues (Hesterberg et al. 2025). Cancer therapies reinforce this trajectory. Radiotherapy and chemotherapy by virtue of their mechanism of action inherently intersect with processes that influence biological aging, including oxidative stress, DNA damage accumulation, cellular senescence as well as chronic inflammatory signaling (Carroll et al. 2022). Underscoring the enduring nature of these adaptations, Scuric et al. (2017) show that increased DNA damage and altered telomerase activity persist in women with breast cancer even 3–6 years following therapy. The resulting accretion of senescent cells disrupt organ homeostasis (Tominaga 2015), while chronic low-grade inflammatory signaling compromises immune competence and metabolic regulation (Frasca and Blomberg 2016; Frasca et al. 2017).

Collectively, tumor and anticancer therapy exposure converge on shared biological aging pathways, embedding accelerated aging as a core component of survivorship physiology.

### Metabolic scars that persist after cancer

The systemic metabolic consequences of cancer likewise extend well beyond the period of active disease. In addition to exhibiting features of accelerated biological aging, many survivors carry persistent “metabolic scars” characterized by long-lasting disruptions in tissue integrity, nutrient handling, and endocrine homeostasis. Among the most clinically significant of these are sustained skeletal muscle wasting and adipose tissue dysfunction, hallmarks of CAC that can persist for years after diagnosis and treatment completion (Bradshaw 2024; Osaki et al. 2024). Consistent with this, elevated inflammatory signaling has been detected long after therapy cessation (Collado-Hidalgo et al. 2006; Ramsey et al. 2006; van der Willik et al. 2018), suggesting that chronic low-grade inflammation may perpetuate systemic metabolic dysfunction even in the absence of active malignancy. Cancer therapies frequently exacerbate these effects, further accelerating tissue degeneration and promoting long-term sarcopenia and frailty (Ness et al. 2015; Jang et al. 2020; Di Meglio and Vaz-Luis 2024). These alterations are not merely cosmetic or functional complications, but reflect durable physiological remodeling with substantial clinical consequences. Young survivors with reduced muscle mass experience fatigue, impaired physical function, and overall reduced quality of life (Pranikoff et al. 2022), while sarcopenic obesity, defined as reduced muscle function along with increased fat mass, increases mortality and risk for ICU admissions (Liu et al. 2024). Current interventions remain limited in their ability to fully reverse these long-term metabolic consequences. Nutritional support and dietary interventions provide partial benefit in some survivors (Rock et al. 2012; Ryding et al. 2025), yet their effectiveness is often constrained by persistent alterations in nutrient metabolism, appetite regulation, and food tolerance that arise during and after treatment (Mayland et al. 2005; Coa et al. 2015; Arends 2018). Consequently, increasing attention has turned toward therapeutic strategies targeting the molecular drivers of cachexia and systemic metabolic dysfunction. One promising example is ponsegromab, a humanized monoclonal antibody targeting GDF-15, a cytokine that suppresses food intake and body mass through signaling via glial cell-derived neurotrophic factor family receptor α-like (GFRAL) in the hindbrain (Wang et al. 2021). Recent clinical studies have demonstrated encouraging effects of ponsegromab on body weight, body composition, and patient quality of life in cachectic cancer (Groarke et al. 2025), highlighting the potential for mechanistically guided interventions aimed at reversing the long-term systemic consequences of tumor–host metabolic reprogramming.

Beyond tissue composition, endocrine and metabolic regulation remains chronically altered. Radiation exposure causes growth hormone (GH) deficiency occurs in >50% of the patient population with childhood brain tumors (Schmiegelow et al. 2000). Hypothyroidism although not generally associated with radiotherapy and chemotherapeutics is commonly seen with treatment with immune checkpoint inhibitors (Wright et al. 2021). Disruption of hormonal axes can often result in major shifts in systemic metabolic regulation. A marquee example of this is insulin sensitivity in the survivor population (Sahinoz et al. 2022; Màrmol et al. 2023). For example, l-asparaginase impairs insulin synthesis and causes persistent hyperglycemia (Whitecar et al. 1970; Panigrahi et al. 2016). Moreover, accelerated tissue aging can also directly impair insulin secretion in β islet cells in the pancreas as well as dysregulate insulin sensitivity through unresolved inflammatory signaling (Shoelson et al. 2006; Zhang et al. 2023). Consistent with this, radiation has been shown to increase the risk for both type 1 and type 2 diabetes in survivors (Friedman et al. 2018; Friedman et al. 2020). Moreover, the use of steroids in the clinic to alleviate treatment side effects are also often associated with insulin resistance (Friedman et al. 2019) and increased progression to diabetes (Majhail et al. 2009). Encouragingly, persistent exercise and metformin partially restore metabolic control in survivors, at least in part by improving insulin management and inflammatory signaling (Meyerhardt et al. 2020; Wang et al. 2020). Additionally, systemic lipid metabolism is also affected. Cancer survivors often present atherogenic lipid profiles impinging on dominance of higher total LDL cholesterol irrespective of the treatment regime, contributing to cardiovascular risk (Ormazabal et al. 2018; Giskeødegård et al. 2022; Kong et al. 2022). Treatment toxicities amplify this burden. Indeed, cisplatin-based therapy has been shown to induce hypomagnesaemia (Scott et al. 2015), a metabolic condition that increases risk for hypertension and cardiovascular diseases (Nielsen 2024). Similarly, anthracycline treatment damages cardiac myocytes directly causing cardiac damage and consequently increasing heart failure incidence in survivors that were exposed to these treatment modalities (Geisberg and Sawyer 2010; Vo et al. 2024). Statins have been shown to mitigate some of these toxicities (Felix et al. 2024), however persistent hepatic drug metabolism alterations in the survivor population have a lasting impact on liver metabolism affecting drug efficacy and safety (Ricart 2017).

Collectively, the loss of tissue integrity and the disruption of metabolic hemostasis constitute an interdependent domain dictating metabolic disease risk in cancer survivors where cancer and treatment history can simultaneously compromise endocrine, muscular and cardiovascular function and where pharmacological interventions can be clinically challenging.

### Neurocognitive consequences of systemic reprogramming

Neurocognitive impairment represents another long-term manifestation of systemic tumor effects. Cancer related cognitive impairment (CRCI) affects attention, memory and processing speed (Wefel et al. 2015), and in severe cases can be accompanied by cerebral atrophy including severe hippocampal atrophy (Karschnia et al. 2025). Multiple mechanisms converge to produce this phenotype. One emerging framework is that tumors and cancer therapies accelerate neuroimmune aging pathways that predispose the brain to chronic dysfunction. Tumor-induced systemic immune aging, observed for example in lymphoma patients (Hesterberg et al. 2025), may promote aging-associated microglial states that contribute directly to neurodegeneration and impaired neuronal homeostasis (Gulen et al. 2023; Jang et al. 2026). Superimposed on this, chemotherapy and radiation therapy exert direct neurotoxic effects that further disrupt neuronal integrity and cognitive function (Dropcho 2010; Was et al. 2022). Importantly, many of these mechanisms are sustained by persistent systemic inflammation, which has been repeatedly associated with long-term neuroinflammatory states in cancer survivors (Schoenberg et al. 2023). Indeed, cognitive impairments remain detectable decades after treatment completion and correlate with circulating inflammatory markers, supporting a model in which unresolved systemic inflammation acts as a chronic driver of CRCI (Koppelmans et al. 2012; van der Willik et al. 2018; Yang and Hendrix 2018).

Metabolic dysregulation may provide an additional mechanistic bridge linking systemic tumor–host interactions to neurocognitive decline. As discussed previously, cancer-associated inflammation profoundly alters tryptophan metabolism through activation of the kynurenine pathway. Within the central nervous system, accumulation of kynurenine-derived metabolites such as quinolinic acid can shift the brain toward a proinflammatory and excitotoxic state through excessive NMDA receptor activation (Garrison et al. 2018). Activated microglia are a major source of quinolinic acid, positioning neuroimmune-metabolic interactions at the center of CRCI pathophysiology. Similarly, elevated circulating lactate, a hallmark of glycolytic tumors during active disease and a feature that can persist in the posttreatment metabolic state (Jones et al. 2019; Broskey et al. 2024), has been shown to promote proinflammatory microglial activation through TLR4 signaling (Yang et al. 2025b). Together, these observations suggest that persistent systemic metabolic abnormalities can directly reprogram neuroimmune function and sustain long-term cognitive dysfunction even after tumor eradication.

Modern cancer therapies themselves can also induce durable neurological toxicity. Immune checkpoint blockade has been associated with long-term neurocognitive and neurological complications in patients (Blansfield et al. 2005; Tarhini 2013; Fletcher and Johnson 2024). Experimental studies further demonstrate that combined PD-1/CTLA-4 inhibition can impair synaptic integrity, activate microglia, and disrupt hippocampal-dependent cognitive function even in tumor-free settings, suggesting that immune hyperactivation alone is sufficient to perturb CNS homeostasis (Ifejeokwu et al. 2025). Likewise, adoptive cell therapies such as CAR-T therapy can trigger severe neurological complications, including immune effector cell-associated neurotoxicity syndrome (ICANS) (Morris et al. 2022; Ren et al. 2024). Emerging evidence suggests that ICANS is not solely an immune-mediated complication, but also reflects widespread metabolic dysregulation within the central nervous system. Inflammatory activation of microglia during ICANS drives extensive rewiring of amino acid metabolism, particularly through activation of the tryptophan–kynurenine pathway, resulting in the accumulation of neuroactive metabolites such as quinolinic acid together with elevated glutamate levels (Santomasso et al. 2018; Xiao et al. 2021). These metabolites promote excitotoxic neuronal injury through excessive NMDA receptor activation while simultaneously reinforcing neuroinflammatory signaling. Thus, CAR-T-associated neurotoxicity illustrates how therapy-induced immune activation can secondarily reshape CNS metabolism, linking systemic inflammatory responses to metabolic mechanisms of neuronal dysfunction and injury.

Importantly, several studies demonstrate an association between cognitive dysfunction and the development of cardiovascular risk factors (Yaffe et al. 2004; Bokura et al. 2010; Muller et al. 2010). Alterations in the CNS have been observed in people with cardiovascular disease risk, including decreased global brain volume (Tiehuis et al. 2014), periventricular white matter hyperintensities (Bokura et al. 2008) and elevated CSF volume (Yau et al. 2012). Conversely, metabolic therapies such as metformin and statins show neuroprotective effects (van der Most et al. 2009; Campbell et al. 2018; Reed et al. 2025), underscoring the critical intersection of cardiometabolic and neuronal health in the survivorship continuum. Rehabilitation therapies and cognitive training offer meaningful benefit (Miladi et al. 2019; Wolfe et al. 2025; Yang et al. 2025a) and preventive strategies like hippocampal-sparing radiation may reduce injury (Weiner 2020).

Collectively, these findings support a conceptual framework in which neurocognitive dysfunction in cancer survivorship arises from persistent systemic tumor–host reprogramming that converges on the brain through inflammatory, immune, and metabolic pathways. In this model, the CNS is not an isolated bystander to cancer progression and treatment, but rather an integrated target of organism-wide physiological remodeling whose long-term disruption contributes substantially to morbidity and reduced quality of life in survivors.

## Conclusion and future perspectives

More than a century ago, observations that one tumor could influence another within the same organism suggested that cancer operates systemically (Enrlich and Apolant 1905). Modern evidence now extends this principle into a new paradigm that tumors reshape host physiology across organs, producing lasting effects that persist after treatment. Cachexia, cardiovascular disease, immune aging, and neurocognitive decline are therefore not unrelated complications but coordinated consequences of tumor-driven macroenvironmental reprogramming. These effects vary by tumor genotype, cancer type, and host physiology (Tan et al. 2012; Chen et al. 2019; Iyengar et al. 2023; Mariean et al. 2023), emphasizing the need to understand cancer as a dynamic interaction between tumor and host over time. Therapeutically targeting tumor-induced host alterations offers opportunities to improve both survival and quality of life (Neyroud et al. 2023). However, more research is required to dissect the intricate complexities of tumor–host interactions that are evidently bi-directional and dynamic in nature. Future research should therefore focus on defining how tumor–host interactions evolve, distinguishing transient responses from stable physiological rewiring, and integrating survivorship biology into therapeutic design. By tracking systemic metabolic and immune trajectories across the cancer continuum, we can move from reacting to late effects toward preventing them and thereby transforming survivorship care into an extension of cancer therapy rather than its aftermath.
